# ^15^N tracing reveals preference for different nitrogen forms of *Fusarium oxysporum* f. sp. *cubense* tropical race 4

**DOI:** 10.3389/fmicb.2023.1102720

**Published:** 2023-02-03

**Authors:** Chen He, Zhongjun Jia, Pingshan Fan, Yunze Ruan, Ye Liang, Jingjing Ma, Jinku Li

**Affiliations:** ^1^College of Tropical Crops, Hainan University, Haikou, China; ^2^State Key Laboratory of Black Soils Conservation and Utilization, Northeast Institute of Geography and Agroecology, Chinese Academy of Sciences, Changchun, China; ^3^Institute of Soil Science, Chinese Academy of Sciences, Nanjing, China

**Keywords:** ^15^N tracing technology, preference, *Foc* TR4, nitrogen forms, banana

## Abstract

Plant uptake of nitrogen is often associated with increased incidence of banana Fusarium wilt, a disease caused by the soil-borne fungus *Fusarium oxysporum* f. sp. *cubense* tropical race 4 (*Foc* TR4). However, the nitrogen metabolic preferences of *Foc* TR4 pathogens remain unknown. In this study, we investigated the ecophysiological patterns of *Foc* TR4 grown on different combinations of organic and inorganic nitrogen. Potato Dextrose Agar (PDA) and Rose Bengal Medium (RBM) were used as an organic nitrogen source, which was sequentially replaced with inorganic N (0, 50% or 90%) in the form ^15^NH_4_NO_3_ or NH_4_^15^NO_3_ to reveal preferential assimilation of ammonium or nitrate. The results showed that mycelium biomass and nitrogen content decreased significantly, while the carbon content and C:N ratio increased in *Foc* TR4 grown on media containing inorganic nitrogen sources. Mycelium biomass was negatively correlated with C:N ratio. Mycelium ^15^N abundance increased significantly between the PDA50 + A50/RBM50 + A50 treatments (50% organic nitrogen+50%^15^NH_4_NO_3_) and the PDA10 + A90/RBM10 + A90 treatments (10% organic nitrogen+90%^15^NH_4_NO_3_). These results indicate that the higher C:N ratio reduced mycelium growth by reducing its biomass and diameter and showed that *Foc* TR4 preferred to use ammonium nitrogen to promote the growth. These findings suggest that treating banana crops with a combination of organic and inorganic (i.e., nitrate) nitrogen could be a better way to defend against Fusarium wilt of banana.

## 1. Introduction

**Figure 1:**
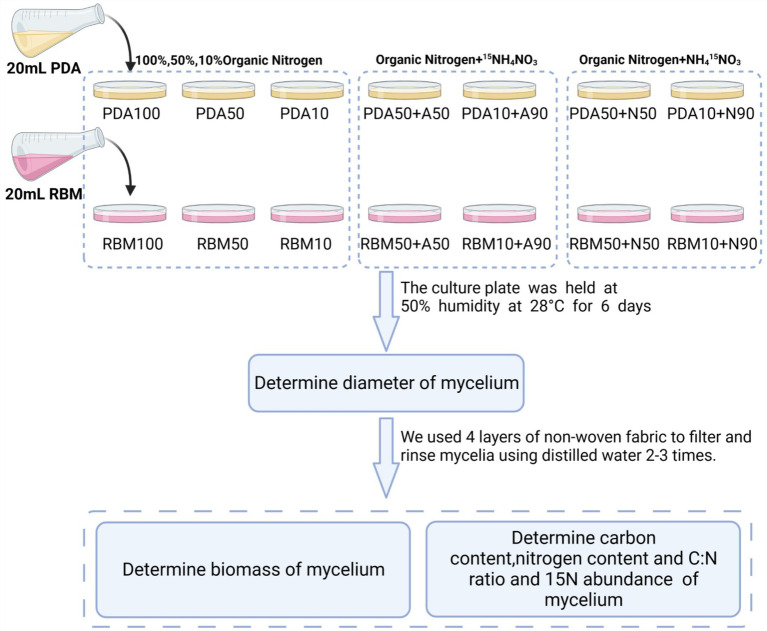
Overview of the experimental design.

Fusarium wilt of banana (FWB), induced by the soil-borne fungus *Fusarium oxysporum* f. sp. *cubense* tropical race 4 (*Foc* TR4), is a serious disease affecting bananas that damages the vascular system and induces cell necrosis to reduce crop yield (Butler, 2013). *Foc* TR4 is highly pathogenic and can infect almost species of banana (Dita et al., 2018). The overuse of chemical nutrients, especially nitrogen (N), exacerbates disease resistance in *Foc* TR4. N is an important nutrient for plant growth and has long been known to play a key role in plant diseases (Fagard et al., 2014). Researchers have demonstrated that fungal pathogens need a high supply of N, but that N inputs also improve plant defense (Tavernier et al., 2007). However, excessive application of N fertilizer improves the availability of nitrogenous compounds for use by pathogens, exacerbating plant infections (Segura et al., 2021). Nitrate (NO_3_^−^) and ammonium (NH_4_^+^) are the two main forms of inorganic nitrogen absorbed and utilized by plants, and each has different effects on the occurrence and control of Fusarium wilt (Mur et al., 2017). Inorganic nutrients play an important role in the occurrence and development of many fungal and bacterial diseases (Walters and Bingham, 2007), and many of these nutrients can inhibit the occurrence of fungal diseases and mitigate the damage they cause (Deliopoulos et al., 2010). Previous work has shown that *Foc* number is positively correlated with NH_4_^+^ content, but negatively correlated to NO_3_^−^ content (Zhou et al., 2017). Current evidence suggests that nitrate fertilizer reduces Fusarium wilt severity, whereas ammonium increases it (Wang et al., 2016). This relationship between N form and disease progression may be due to the acidifying effect of ammonium fertilizer, as soil acidification increases FWB severity (Teixeira et al., 2021; Segura et al., 2022). Fusaric acid (FA), the main fungal toxin produced by *Foc* TR4, is critical for FWB development (Wang et al., 2012). Nitrate increases organic acid metabolism and related gene expression, ammonium increases amino acid metabolism and related gene expression, resulting in amino acid accumulation in cucumber plants. This further stimulates FA production and *Foc* sporulation, increasing disease incidence (Wang et al., 2019). Moreover, previous studies have shown that mycelium growth can occur when amino acids are the only available N source (Solomon and Oliver, 2002). At present, there is not a universally applicable method for controlling FWB due to differences in how different soils affects *Foc* TR4’s physiological characteristics. The application of inorganic nitrogen fertilizer causes soil acidification, which may explain the frequent occurrence of banana soil-borne Fusarium wilt. Previous studies have focused mostly on the effect of different nitrogen forms on *Foc* TR4, but none have investigated which form of nitrogen is preferred by the pathogen. Therefore, it is important to investigate *Foc* TR4’s response mechanism to nitrogen fertilization in terms of physiological characteristics (Figure 1).

## 2. Materials and methods

### 2.1. Experimental design

This experiment was conducted in 2021 at Hainan University (Haidian Campus). ^15^NH_4_NO_3_/NH_4_^15^NO_3_ was added to Potato Dextrose Agar (PDA) and Rose Bengal Medium (RBM) to prepare the new culture medium with three replicates for each of the following composition: PDA100 (100% organic nitrogen), PDA50 (50% organic nitrogen), PDA50 + A50 (50% organic nitrogen+50%^15^NH_4_NO_3_), PDA50 + N50 (50% organic nitrogen+50% NH_4_^15^NO_3_), PDA10 (10% organic nitrogen), PDA10 + A90 (10% organic nitrogen+90%^15^NH_4_NO_3_), PDA10 + N90 (10% organic nitrogen+90%NH_4_^15^NO_3_), RBM100 (100% organic nitrogen), RBM50 (50% organic nitrogen), RBM50 + A50 (50% organic nitrogen+50%^15^NH_4_NO_3_), RBM50 + N50 (50% organic nitrogen+50% NH_4_^15^NO_3_), RBM10 (10% organic nitrogen), RBM10 + A90 (10% organic nitrogen+90%^15^NH_4_NO_3_), RBM10 + N90 (10% organic nitrogen+90%NH_4_^15^NO_3_). 20 mL culture solution and ^15^NH_4_NO_3_ / NH_4_^15^NO_3_ solution were added to each plate, and each treatment had three replicates. *Foc* TR4 mycelium with the same growth and thickness as the culture medium was prepared by using a 1 cm diameter punch and was used to inoculate the new culture plate. The culture plate was held at 50% humidity at 28°C for 6 days. Media composition is presented in Table 1. A total of 1 L of culture medium was autoclaved at 121°C for 20–30 min. 0.40 g ^15^NH_4_NO_3_ / NH_4_^15^NO_3_ (abundance of ^15^N:99%) was mixed with 10 ml of sterile water, and 0.04 g/ml ^15^NH_4_NO_3_ and NH_4_^15^NO_3_ solution were prepared. Organic nitrogen (PDA and RBM) was added to each culture medium listed in Table 2.

**Table 1:** Medium composition.

Culture medium	Composition	Dosage (g)
Potato Dextrose Agar	Potato extract powder (Total N ≥ 8%)	5.000
	D-Glucose	20.000
	Agar	16.000
Rose Bengal Medium	PEPTONE (Total N ≥ 13%)	5.000
	KH₂PO_4_	1.000
	MgSO_4_	0.500
	Agar	16.000
	D-Glucose	10.000
	Rose Bengal	0.033
	Chloramphenicol	0.100

**Table 2:** Addition of nitrogen in each culture medium.

Culture medium	Treatment	Organic nitrogen (g)	^15^NH_4_NO_3_ (mL)	NH_4_^15^NO_3_
				(mL)
Potato Dextrose Agar	PDA100	≥0.008	–	–
	PDA50	≥0.004	–	–
	PDA50 + A50	≥0.004	0.290	–
	PDA50 + N50	≥0.004	–	0.290
	PDA10	≥0.0008	–	–
	PDA10 + A90	≥0.0008	0.515	–
	PDA10 + N90	≥0.0008	–	0.515
Rose Bengal Medium	RBM100	≥0.013	–	–
	RBM50	≥0.0065	–	–
	RBM50 + A50	≥0.0065	0.465	–
	RBM50 + N50	≥0.0065	–	0.465
	RBM10	≥0.0013	–	–
	RBM10 + A90	≥0.0013	0.840	–
	RBM10 + N90	≥0.0013	–	0.840

We observed the growth of *Foc* TR4 mycelia, taking pictures and measuring growth after 2, 4, and 6 days. After the sixth day, we used four layers of non-woven fabric to filter and rinse mycelia using distilled water 2–3 times. Samples were dried in an oven at 105°C for 24 to a constant weight. Biomass was determined, after which mycelia were ground and crushed before being placed in a 5 ml test tube. Samples were sent to the Element and Stable Isotope Laboratory of the Analytical and Testing Center at Hainan University to determine the carbon content, nitrogen content, and ^15^N abundance using an isoprime precision mass spectrometer (Germany).

### 2.2. Statistical analyses

Microsoft Excel 2010 was used to organize the data and SPSS 24.0 software was used for statistical analysis of the data. One-way analysis of variance (ANOVA) and least significant difference (LSD) tests (*p* < 0.05) were used for multiple comparison analyses. Graphs were generated on Origin 2018.

## 3. Results

**Figure 2:**
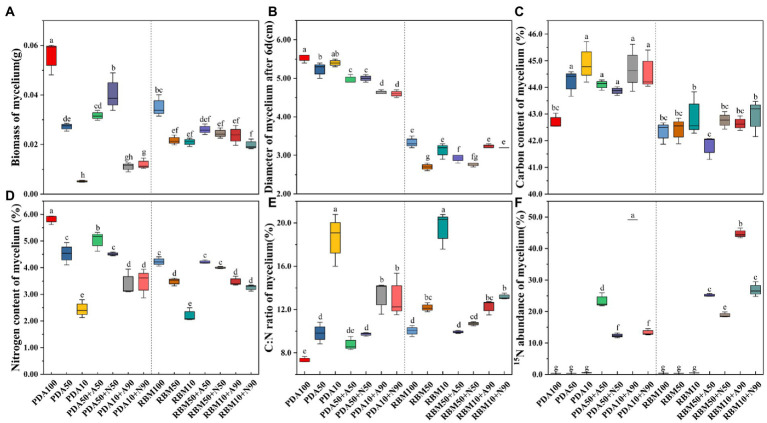
*Foc* TR4 prefers using ammonium to nitrate for growth when organic N is limited. Mycelium biomass on PDA and RBM **(A)**. Mycelium diameter on PDA and RBM **(B)**. Mycelium carbon content on PDA and RBM **(C)**. Mycelium nitrogen content on PDA and RBM **(D)**. Mycelium C:N ratio on PDA and RBM **(E)**. Mycelium ^15^N abundance on PDA and RBM **(F)**. Error bars represent standard errors (*n* = 3). Different letters indicate significant differences across different treatments (*p*<0.05).

In addition to being significantly affected by nitrogen form, mycelium biomass and diameter were closely related to nitrogen uptake and utilization. Compared with the control (PDA100), biomass decreased significantly on PDA and RBM (*p* < 0.05). Biomass of the combined organic and inorganic nitrogen treatment was significantly lower than that of the pure organic nitrogen treatment (PDA100 or RBM100) on PDA and RBM. *Foc* TR4 preferentially utilized organic nitrogen rather than inorganic nitrogen (Figure 2A). In the combined organic and inorganic nitrogen treatment, mycelial diameter was higher on PDA compared to RBM (Figure 2B), indicating PDA is preferred for *Foc* TR4 growth. The carbon content, nitrogen content, and C:N ratio significantly influenced fungal growth. The mycelial carbon content in experimental treatments was 3.55, 3.28, 2.72, 5.13, 4.66, and 4.32% higher than the control. All experimental groups exhibited higher carbon content relative to the control, except the RBM50 + A50 group, which was 1.27% lower; however, differences between treatments within the RBM group were not significant (Figure 2C). In contrast, nitrogen content decreased on both PDA and RBM. On PDA, nitrogen content was 22.24, 13.53, 22.53, 58.09, 41.79, and 40.38% lower than in the control treatment (Figure 2D), suggesting that *Foc* TR4 increased carbon uptake and use in response to poor nitrogen utilization. The C:N ratio is an important indicator of nutrient utilization, and both high and low C:N ratios are detrimental to cell growth and exogenous protein expression and accumulation. The C:N ratios of mycelia in the experimental group increased significantly compared to the control group. The C:N ratios of mycelia in PDA10 + A90 and PDA10 + N90 treatments were 81.60 and 77.73% higher, respectively, than that of the control on PDA (*p* < 0.05). In addition, the C:N ratio in mycelia grown on RBM exhibited a similar pattern (Figure 2E). In the ^15^N abundance experiment, tracer uptake was higher on PDA50 + A50 than on PDA50 + N50 and higher in PDA10 + A90 than in PDA10 + N90, with rates of uptake 88.63 and 267.53% higher, respectively, than growth on PDA (*p* > 0.05). A similar pattern was observed in mycelia grown on RBM, suggesting that *Foc* TR4 prefers to use ammonium as a nitrogen source over nitrate when organic nitrogen is limited (Figure 2F).

**Figure 3:**
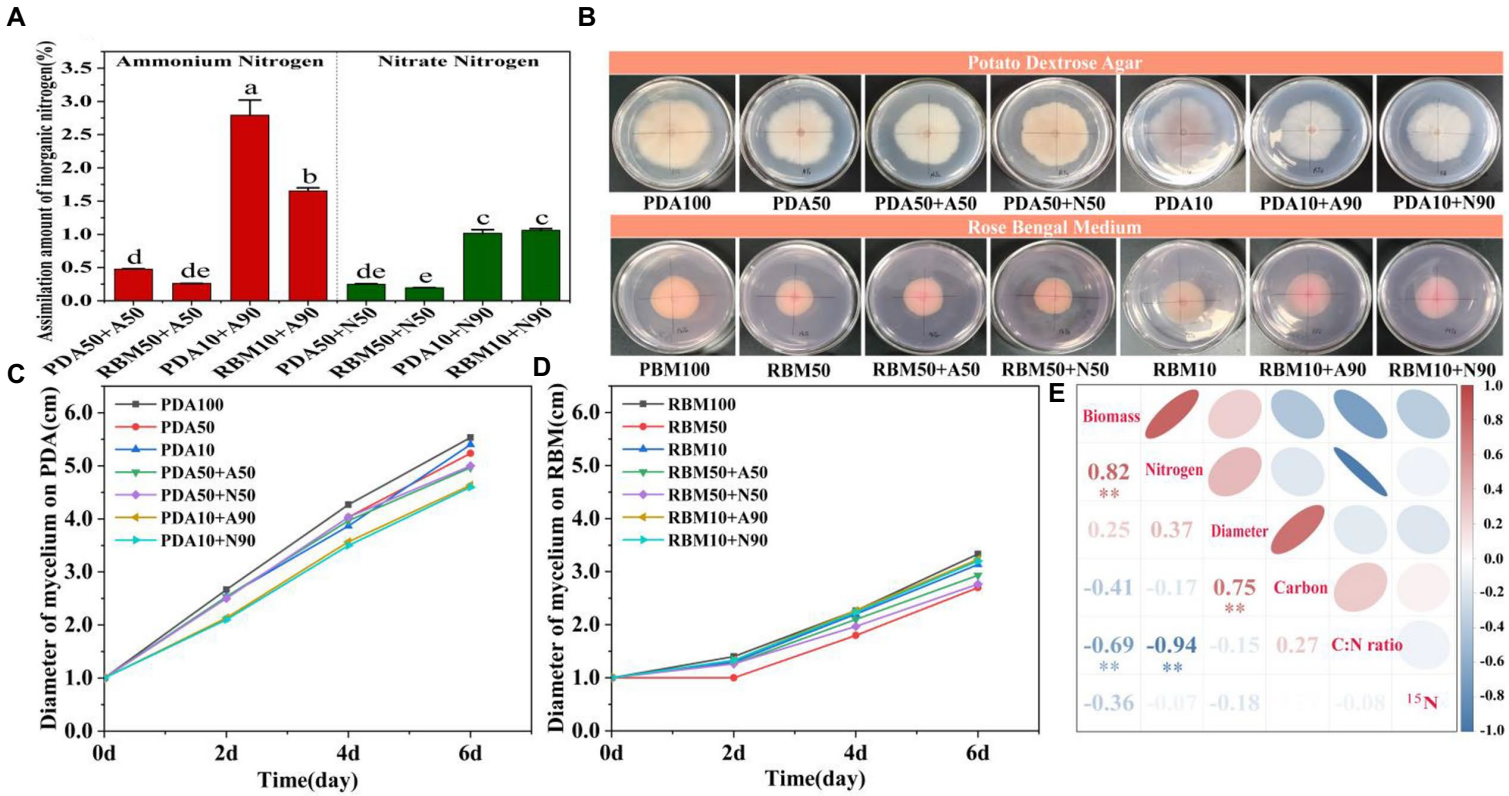
Inorganic nitrogen assimilation on PDA and RBM **(A)**. Mycelium diameter 6 days post-inoculation on PDA and RBM **(B)**. Change in mycelium diameter during incubation on PDA **(C)**. Change in mycelium diameter during incubation on RBM **(D)**. Pearson correlation analyses of biomass, diameter, nitrogen content, ^15^N abundance, carbon content, and C:N ratio **(E)**. Blue indicates negative correlation, and red indicates positive correlation. Smaller shape and darker color indicates larger correlation coefficients, ** indicates *p* < 0.01.

We found that ammonium and nitrate assimilation was significantly higher in the 90% organic nitrogen reduction treatment compared to the 50% nitrogen reduction treatment; moreover, assimilation of inorganic nitrogen was higher in the PDA10 + 90 group than in all other treatments, indicating that reducing organic nitrogen availability increases the assimilation efficiency of inorganic nitrogen, especially ammonium (Figure 3A). Mycelium diameter was notably different between PDA and RBM treatments (Figure 3B). As the experiment proceeded, mycelium diameter gradually increased across all treatments, but the pattern on increase varied. For *Foc* TR4 grown on PDA, mycelium diameter increased rapidly after inoculation (Figure 3C); a similar rapid increase in mycelium diameter was observed for RBM, but not until the second day of growth (Figure 3D). Pearson correlation analysis showed that the biomass of mycelium was significantly correlated with nitrogen content and C:N ratio. Mycelium biomass was positively correlated with nitrogen content, but negatively correlated with C:N ratio. This suggests that the C:N ratio is one of the most important factors affecting the growth of *Foc* TR4. Carbon content was also significantly correlated with mycelial diameter (Figure 3E).

## 4. Discussion

Fusarium wilt of banana is one of the most devastating soil-borne fungal diseases in banana production in the world. Nitrogen assimilation is necessary for the health growth of plants and strongly influences disease development and plant resistance. Previous work has found that nitrogen form influences the development and progression of Fusarium wilt disease (Gu et al., 2020). Pathogenic fungus can assimilate nitrogen occurring in different forms, such as ammonium nitrogen, nitrate nitrogen, and amino acids (Mur et al., 2017). Previous studies have shown that mycelium growth can occur when amino acids are the sole source of nitrogen (Solomon and Oliver, 2002). Zhou et al. (2019) showed that the content of total organic carbon, total organic nitrogen and available potassium in soil with blight was significantly higher than in healthy soil. Studies have shown that organic nitrogen sources such as beef extract and peptone promote the germination of cucumber wilt spores more effectively (Zhang et al., 2011). Bitter melon wilt can utilize multiple nitrogen sources, including ammonium and nitrate in organic nitrogen and inorganic nitrogen. However, the type of nitrogen required is differs across developmental and growth stages, and peptone is the form that most effectively promotes mycelial growth (Xiao et al., 2008). In this study, Fusarium wilt of banana preferentially used organic nitrogen over inorganic nitrogen, indicating that organic nitrogen is sufficient to support growth and that the addition of inorganic nitrogen will reduce the pathogen’s growth by reducing its biomass. It is generally understood that enhanced NH_4_^+^ availability increases plant susceptibility by enhancing the availability of apoplastic sugar, amino acids, and g-amino-n-butyric acid to the pathogen (Gupta et al., 2013). Interestingly, in this study, *Foc* TR4 prefered ammonium nitrogen in the presence of organic nitrogen, which is consistent with previous studies. At the same time, due to organic nitrogen limitation, fungal carbon content increased, but nitrogen content decreased, resulting in higher C:N ratios. This result has rarely been reported in previous studies.

## 5. Conclusion

In conclusion, the combination of organic and inorganic nitrogen treatments reduced mycelium biomass and nitrogen content significantly compared to an organic N source (PDA100 or RBM100); mycelium carbon content and C:N ratio increased significantly on PDA and RBM; and the mycelium biomass was negatively correlated with C:N ratio. A significant increase in ^15^N abundance was observed in mycelium grown on PDA50 + A50, PDA10 + A90, RBM50 + A50, and RBM10 + A90. These results indicate that mycelial biomass and diameter decreased as the C:N ratio increased, and that *Foc* TR4 preferentially utilized organic nitrogen over inorganic nitrogen. More importantly, *Foc* TR4 preferred to use ammonium nitrogen over nitrate to promote growth when organic N is limited. Therefore, we hypothesize that excessive application of ammonium nitrogen will aggravate the degree of banana wilt disease, while treating crops with a combination of organic and inorganic nitrogen fertilizers will reduce disease severity. This provides a new idea and reference for using nitrogen fertilization in banana cultivation management in the future.

## Data availability statement

The raw data supporting the conclusions of this article will be made available by the authors, without undue reservation.

## Author contributions

ZJ conceived and designed the experiment. CH, PF, YL, and JL conducted the experiment. ZJ and CH analyzed data. YR contributed to materials and reagents. CH and JM wrote the article with assistance from all coauthors. All authors have read and approved the final manuscript.

## Funding

This study was supported by the Project of Sanya Yazhou Bay Science and Technology City (SCKJ-JYRC-2022-94), the Strategic Priority Research Program of Chinese Academy of Sciences (XDA28020203), and the National Natural Science Foundation of China (91751204, 32160750, and 31672239).

## Conflict of interest

The authors declare that the research was conducted in the absence of any commercial or financial relationships that could be construed as a potential conflict of interest.

## Publisher’s note

All claims expressed in this article are solely those of the authors and do not necessarily represent those of their affiliated organizations, or those of the publisher, the editors and the reviewers. Any product that may be evaluated in this article, or claim that may be made by its manufacturer, is not guaranteed or endorsed by the publisher.
